# The role and therapeutic targeting of the CCL2/CCR2 signaling axis in inflammatory and fibrotic diseases

**DOI:** 10.3389/fimmu.2024.1497026

**Published:** 2025-01-09

**Authors:** Shan Guo, Qi Zhang, Yingjie Guo, Xiaoyan Yin, Peng Zhang, Tao Mao, Zibin Tian, Xiaoyu Li

**Affiliations:** Department of Gastroenterology, The Affiliated Hospital of Qingdao University, Qingdao, China

**Keywords:** CCL2, CCR2, inflammation, fibrosis, immune regulation, clinical diagnosis and treatment

## Abstract

CCL2, a pivotal cytokine within the chemokine family, functions by binding to its receptor CCR2. The CCL2/CCR2 signaling pathway plays a crucial role in the development of fibrosis across multiple organ systems by modulating the recruitment and activation of immune cells, which in turn influences the progression of fibrotic diseases in the liver, intestines, pancreas, heart, lungs, kidneys, and other organs. This paper introduces the biological functions of CCL2 and CCR2, highlighting their similarities and differences concerning fibrotic disorders in various organ systems, and reviews recent progress in the diagnosis and treatment of clinical fibrotic diseases linked to the CCL2/CCR2 signaling pathway. Additionally, further in-depth research is needed to explore the clinical significance of the CCL2/CCR2 axis in fibrotic conditions affecting different organs.

## Introduction

Chemokines are a large family of small proteins that play a crucial role in regulating cell movement (1), particularly in guiding leukocyte migration (2), which is central to maintaining immune system balance (3). In 2000, chemokines were systematically classified into four families: CXC, CC, CX3C, and C (4). Among these, CC chemokine ligand 2 (CCL2), also known as monocyte chemotactic protein-1 (MCP-1), is a key regulator in immune response. Discovered in 1989, CCL2 is primarily anchored to endothelial cell membranes (5) and is most abundantly expressed in monocytes, macrophages, and lymphocytes (6). Other cells, such as smooth muscle cells, endothelial cells, and fibroblasts, can also secrete CCL2 (7), particularly in response to cytokines like interleukin (IL)-6, tumor necrosis factor-α (TNF-α), and transforming growth factor-β (TGF-β) (8).

**Figure 1:**
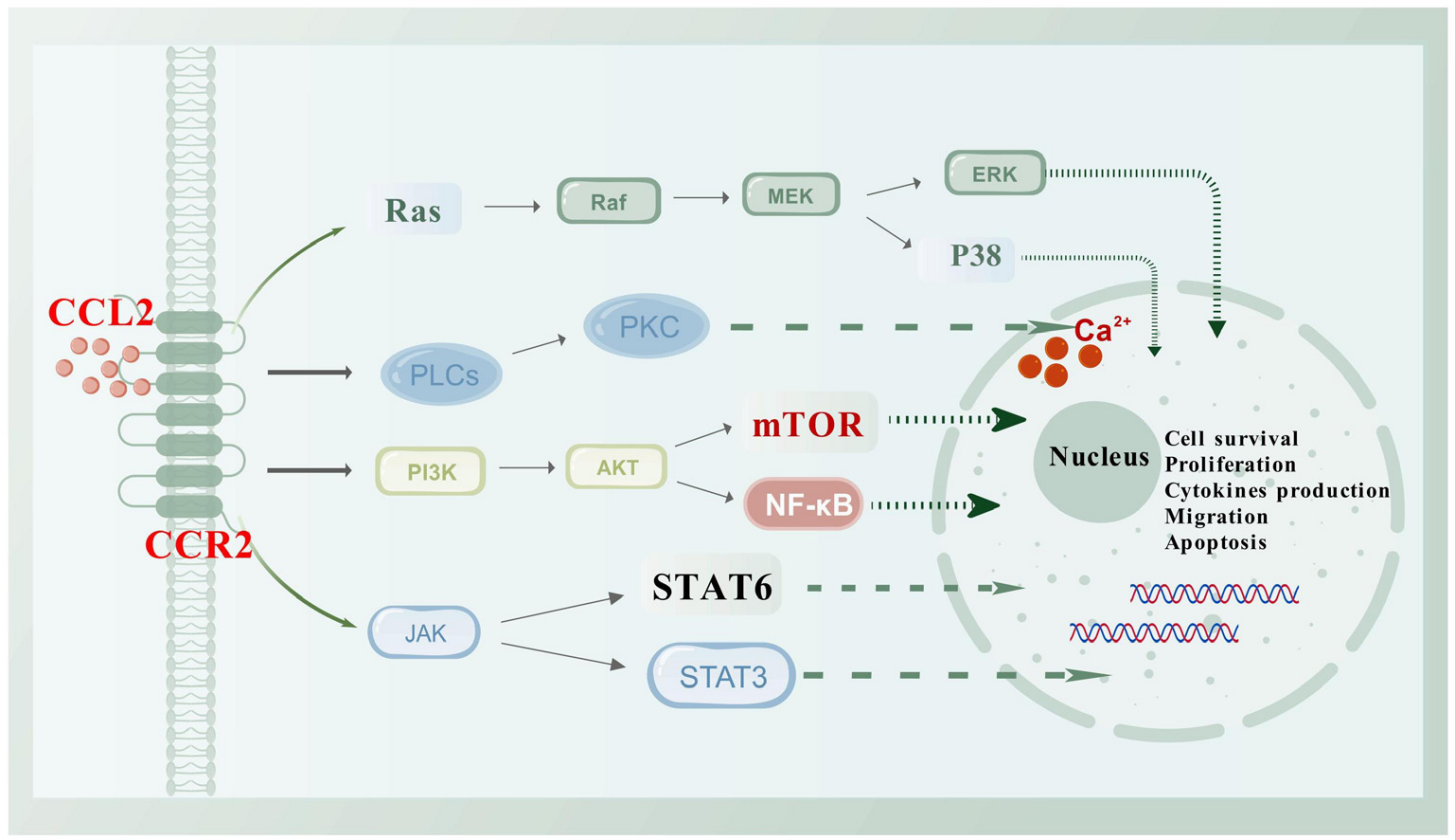
Schematic diagram of the CCL2/CCR2 axis and its associated signaling pathways. CCR2, a classic G protein-coupled receptor, activates a variety of downstream signaling pathways upon binding to its ligand CCL2, such as PI3K/Akt, JAK/STAT, and P38/MAPK. Activation of these pathways leads to the regulation of various transcription factors and genes involved in cell survival, proliferation, cytokine production, migration, and apoptosis. This figure was created with biogdp.com.

CCL2 exerts its effects mainly by binding to the receptor CCR2 (9), which has the highest affinity for CCL2 due to its specific structural characteristics (10). This binding activates various downstream pathways (**Figure 1**), resulting in diverse chemotactic responses, particularly in monocytes. The CCL2-CCR2 interaction not only promotes cell migration but also regulates cell adhesion and macrophage chemotaxis. Upon ligand binding, CCR2 experiences conformational changes that initiate the activation of phospholipase C (PLC). This activation leads to an increased release of Ca^2+^ ions and the subsequent activation of protein kinase C (PKC) and phosphoinositide 3-OH kinase (PI3K). These processes further activate signaling molecules such as protein kinase B (PKB, Akt) and mitogen-activated protein kinase (MAPK). This signaling pathway is crucial for the regulation of central transduction mechanisms within the cell (11–13). Additionally, it plays a significant role in cancer progression by activating pathways such as p38-MAPK (14, 15) and PI3K/AKT/mTOR (16–18), which enhance tumor cell invasion, migration, and survival.

**Figure 2:**
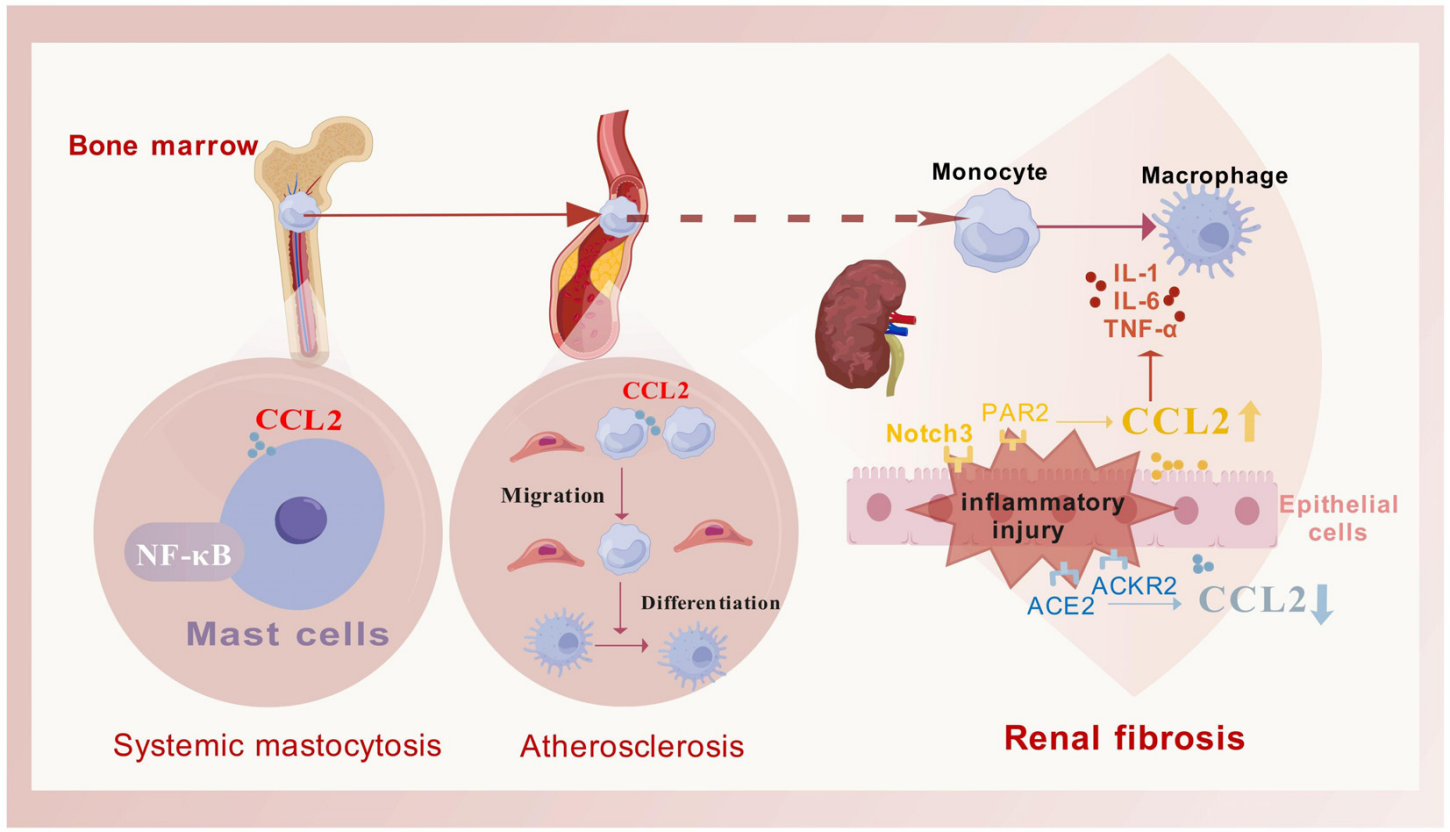
The primary role process of CCL2 in fibrotic diseases. CCL2 facilitates the mobilization of monocytes from the bone marrow into the bloodstream and guides their migration to targeted inflammatory fibrotic areas. Once there, these monocytes differentiate into macrophages to address tissue damage (as exemplified by atherosclerotic plaques), this process is frequently associated with the activation of the NF-κB signaling pathway (as exemplified by systemic mastocytosis). Additionally, the regulation of CCL2 secretion is often influenced by various receptors, which in turn modulate the release of inflammatory mediators (as seen in fibrosis associated with chronic kidney disease). This figure was created with biogdp.com.

CCL2 is extensively involved in disease regulation, particularly in inflammation, fibrosis, and cancer. It mobilizes monocytes from the bone marrow into the bloodstream and directs their migration to sites of inflammation (19), underscoring its essential role in immune defense (20). Beyond inflammation, CCL2 contributes to fibrosis development in various organs like the liver, pancreas, and kidneys (**Figure 2**). It also plays a role in cancer biology, known as the “tumor chemokine” (21), aiding in tumor initiation, growth, and metastasis (22, 23). Tumor-associated macrophages secrete CCL2 (24), which aids in the progression of cancers like breast (25) and pancreatic cancers (26, 27). CCL2 expression in the tumor microenvironment, involving various cell types, highlights its significance in cancer pathology (28) and its potential as a therapeutic target.

Chemokine receptors have been targeted in therapeutic strategies, and recent years have seen promising results from CCL2/CCR2 antagonists in treating inflammatory and fibrotic diseases (29) and cancer immunotherapy (30, 31). This review explores the mechanisms and the diagnostic and therapeutic potentials of the CCL2/CCR2 axis in inflammatory and fibrotic diseases. These conditions are characterized by chronic inflammation leading to fibrosis, where prolonged immune responses cause excessive extracellular matrix deposition, tissue scarring, and organ dysfunction. Understanding the role of the CCL2/CCR2 axis in fibrosis will provide a basis for developing novel therapies.

## Mechanisms of the CCL2/CCR2 axis in inflammatory and fibrotic diseases

CCL2 plays a pivotal role in fibrogenesis in the liver, pancreas, and intestine by attracting immune cells such as monocytes and macrophages to fibrotic areas, promoting inflammatory responses and matrix remodeling. In viral hepatitis, CCL2 helps create an immunosuppressive microenvironment that facilitates fibrosis progression and favors viral infection. In liver fibrosis, CCL2 synergizes with TGF-β to activate hepatic stellate cells (HSCs) and promote matrix deposition. In intestinal fibrosis, CCL2 works with intestinal-specific factors like IL-17. In pancreatic fibrosis, CCL2 leads to M2-like polarization of macrophages after attracting immune cells to the tissue.

## Hepatic fibrosis diseases

CCL2 is crucial in liver diseases such as acute and chronic liver injury, cirrhosis, and tumor progression (32). Liver inflammation often coexists with fibrosis, driven by the recruitment and polarization of macrophages, particularly through hepatic stellate and Kupffer cell activation (33, 34). Key pathways like hedgehog (35) and TGF-β (36) are involved in these processes, contributing to conditions like non-alcoholic steatohepatitis (NASH) and hepatocellular carcinoma (HCC) (37).

### NASH

NASH results from excessive fat accumulation in the liver, causing hepatocyte damage and triggering inflammation. This inflammatory state activates HSCs, leading to fibrosis, cirrhosis, or liver cancer. Studies have shown that increased expression of CCL2 is a hallmark of NASH, promoting the infiltration of monocyte-derived macrophages through CCL2-mediated chemotaxis.

CCL2 is implicated in NASH progression through several mechanisms. Li et al. (38) identified methyltransferase 3 (METTL3) as a key negative regulator, loss of Mettl3 accelerates NASH by enhancing CD36-dependent fatty acid uptake and CCL2-driven inflammation. The absence of CX3CR1 promotes a shift from M1 to M2 macrophages, slowing NASH progression. CCL2 deficiency in *Cx3cr1* ^-/-^ mice reduces macrophage infiltration and supports M2 dominance in the liver, alleviating NASH (39). Recent research has indicated that the activation of Notch signaling pathways in hepatocytes is crucial for the advancement of liver fibrosis linked to NASH (40). Additionally, existing literature has underscored the role of Notch signaling in hepatocytes, which further facilitates the infiltration of macrophages dependent on CCL2, thereby exacerbating fibrosis (41).

Autophagy in liver sinusoidal endothelial cells is also involved in NASH. Insufficient autophagy increases the expression of CCL2, CCL5, and CD68, exacerbating fibrosis in high-fat diet-fed mice (42). CD11c^+^CD206^+^ cells, which express high levels of CCR2, show increased CCL2 expression in NASH, correlating with disease severity. Inhibition of CCR2 reduces the infiltration of CD11b^+^CD11c^+^F4/80^+^ monocytes and improves liver inflammation and fibrosis in NASH models (43).

Multiple signaling pathways are implicated in steatohepatitis, with the CCL2/CCR2 axis playing a key role. Gasdermin D, involved in programmed necrosis, promotes the secretion of pro-inflammatory cytokines IL-1β and CCL2 and activates the NF-κB pathway, driving NASH progression (44). The NLRP3-IL-1β pathway also contributes to inflammation and obesity-related comorbidities. Inhibition of NLRP3 inflammasome reduces lipopolysaccharide-induced inflammation by down-regulating CCL2 mRNA levels (45). Suppressing the IL-33 signaling pathway can inhibit NASH progression by down-regulating CCL2 and α-SMA expression (46), while activating IL-19 signaling reduces HSC migration by down-regulating CCL2 expression, alleviating liver fibrosis (47).

### Viral hepatitis

CCL2 plays a specific role in immune evasion during hepatitis B virus (HBV) infection. Chronic HBV infection, a major risk factor for liver diseases such as HCC, can persist for decades. HBV evades immune response by downregulating CCL2 (48), reducing inflammatory monocyte and macrophage recruitment to the liver. In chronic HBV cases complicated by cirrhosis, plasma CCL2 levels tend to decrease (49). Additionally, HBeAg can promote HSC proliferation, movement, and contraction in a macrophage-dependent manner, inducing CCL2 production that activates HSCs and worsens liver fibrosis (50).

Hepatitis C virus (HCV)-mediated hepatitis is also a significant global health issue, particularly in the United States. Studies show that HCV-infected individuals can downregulate specific microRNAs, such as miRNA-107 and miRNA-449a, to regulate CCL2 expression by targeting the interleukin-6 receptor (IL-6R) complex (51), suggesting potential therapeutic avenues. A novel pathway involving the HCV core protein interacting with gC1qR has been identified, leading to CCL2 and CXCL10 secretion in macrophages via the NF-κB signaling pathway (52).

### Other hepatic fibrotic diseases

Analysis of the liver fibrosis expression dataset GSE84044 from the GEO database identified 10 key genes in the protein interaction network, including CCL2, highlighting its importance in fibrosis and inflammation (53). Glucocorticoid-induced leucine zippers (GILZ), encoded by the *Tsc22d3* gene in mice, mimic glucocorticoids’ anti-inflammatory effects. Mice deficient in GILZ show increased CCL2 production and pro-inflammatory leukocyte infiltration in early liver fibrosis, accelerating its progression (54). Moreover, sphingosine kinase 1 (SPHK1) levels were significantly higher in fibrotic compared to normal human livers. SPHK1 knockout in Kupffer cells reduced CCL2 secretion, while its knockout in HSCs decreased CCR2 expression (55).

CCL2 also has a role in pediatric liver fibrosis. A recent study identified *FOCAD* germline recessive mutation in pediatric liver cirrhosis. In a zebrafish model with *FOCAD* deficiency, liver injury was accompanied by increased CCL2 expression, suggesting that targeting the CCL2/CCR2 axis could be a new approach for treating pediatric liver cirrhosis (56).

## Inflammatory bowel disease

Inflammatory bowel disease (IBD) is a chronic autoimmune inflammatory disease affecting the gastrointestinal tract, with two main subtypes: ulcerative colitis (UC) and Crohn’s disease (CD). The dextran sodium sulfate (DSS)-induced colitis model, which activates the classic NF-κB signaling pathway, is widely used to study UC. In intestinal tissues, CCL2 responds to diverse signals. Inflammatory damage triggers monocytes to secrete CCL2, recruiting white blood cells or macrophages to injury sites, ultimately leading to intestinal fibrosis.

In early intestinal inflammation, Ly6c high-expressing cells markedly enhance the expression of *CCL2* and *CCR2* genes through the activation of STAT1 signaling, in contrast to Ly6c intermediate-expressing cells. Additionally, inhibiting the CCR2 pathway can mitigate colonic damage in models of acute colitis (57). PC3-secreted microproteins (PSMP) upregulate phosphorylated ERK levels via the CCR2 pathway (58), driving CCR2^+^ monocyte migration to inflamed colonic tissue. PSMP attracts Ly6c^hi^ monocytes in a CCR2-dependent manner, aided by Dectin-1 on myeloid cells (59, 60), while cobalt protoporphyrin IX (CoPP) reduces the migration of CCR2^+^ Ly6c^hi^ monocytes to the inflamed colon (61).

As inflammation reaches the muscle layer, Ly6c^+^ monocytes infiltrate, adopting a unique transcriptional state and promoting muscle inflammation. They alter the microenvironment, promoting more monocyte infiltration, which then differentiates into anti-inflammatory CD206^+^ macrophages through CCL2 (62). Bone marrow-derived mesenchymal stromal cells (BM-MSCs) secrete CCL2, influencing colitis development, while IL-10 in MSCs polarizes resident macrophages (63). During chronic inflammation, CCR2^+^ monocytes and fibrocytes infiltrate the colon, promoting fibrosis by inhibiting collagen degradation (64).

CCL2 promotes intestinal fibrosis by activating pro-inflammatory factors, notably IL-6, a process reversible with CCR2 antagonist RS102895 (65). Antisense IL-7 (IL-7-AS) accelerates inflammation by upregulating IL-6 and CCL2 (66). Poly(rC)-binding protein 1 (PCBP1) deficiency reduces CCL2 and IL-6 production in colitis macrophages (67). Additionally, in colitis models, CCR2 and CD30L expression in monocytes are positively correlated; CD30L drives monocyte homing and differentiation via the CCL2/CCR2 axis and NF-κB pathway, enhancing inflammation (68).

CCL2 is also involved in hyperoxia-induced intestinal injury. IL-17D, a member of the IL-17 family, promotes CCL2 expression in intestinal epithelial cells under hyperoxic conditions, leading to chronic intestinal inflammation (69).

## Chronic pancreatitis

Chronic pancreatitis (CP) is characterized by progressive, irreversible inflammation and fibrosis, with pancreatic stellate cells (PSCs) playing a key role in this process (70). Studies in CP patients have shown that prostaglandin E2 mediates CCL2 synthesis in PSCs via TNF-α regulation, and inhibiting cyclooxygenase (COX)-2 activity can slow the progress of pancreatitis and fibrosis (71).

Research on CCL2’s role in CP progression is limited. Interestingly, alternatively activated macrophages (M2) secrete PDGF and TGF-β, which activate PSCs and promote fibrosis—a key factor in CP development (72). This contrasts with the immune microenvironment in NASH (39), highlighting the plasticity of macrophages and suggesting that CCL2 regulates immune cells differently across organs. Additionally, during chronic pancreatic inflammation, CCL2 secretion and NF-κB pathway activation persist (73).

## Cardiovascular fibrotic diseases

Cardiovascular diseases remain a leading cause of morbidity and mortality worldwide (74). Recent studies highlight the inflammatory immune response as a central mechanism in their pathogenesis (75). Among the key mediators, CCL2 plays a crucial role in regulating leukocyte migration and infiltration in various cardiovascular diseases. Consequently, CCL2/CCR2 inhibitors are being explored as novel therapeutic targets for inflammation-related cardiovascular conditions (76).

### Myocardial infarction

During the healing process of Myocardial infarction (MI), macrophages undergo dynamic polarization. CCL2 is differentially expressed in LPS-induced M1 and IL-4-induced M2 macrophages (77). Unlike in the liver and pancreas, CCL2 deficiency in the heart results in reduced total macrophage and M1 macrophage numbers in the infarcted area, while M2 macrophages increase (78). This suggests that CCL2 is a pivotal regulator of macrophage polarization during MI healing, both *in vivo* and *in vitro*, and it specifically promotes M1 polarization. The organ-specific and environment-dependent nature of CCL2’s role likely explains these differential effects.

The repair process following MI is critical for cardiac recovery. In response to acute myocardial ischemia-reperfusion (MIR), macrophages infiltrate damaged heart tissue and shift their polarization to manage acute inflammation and subsequent fibrotic remodeling. Studies indicate that CCL2/CCR2 signaling in macrophages facilitates the transition from acute injury to chronic fibrosis in MIR in mice through the NLRP3 inflammatory pathway and phenotypic changes (79). Furthermore, IL-34 enhances CCL2 expression by maintaining NF-κB pathway activation, aggravating cardiac remodeling and fibrosis after reperfusion injury (80).

### Other cardiovascular inflammatory diseases

A meta-analysis by Georgakis et al. demonstrated that circulating CCL2 levels, synonymous with CCL2, are strongly associated with cardiovascular mortality (81). Elevated CCL2 levels in atherosclerotic plaques correlate with increased plaque vulnerability, and persistently high circulating CCL2 levels heighten the risk of adverse outcomes following plaque removal (82). Moreover, circulating CCL2 levels appear genetically influenced; higher levels are linked to a greater risk of stroke, suggesting that CCL2-targeted therapies might reduce stroke incidence (83).

CCL2 is also implicated in the regulation of circadian rhythms during atherosclerosis progression. Winter et al. (84) revealed that CCL2 is a key chemokine at atherosclerotic sites and that the chronic inflammation of large blood vessels depends on the rhythmic release of CCL2, which peaks in the early stage and declines later. This discovery opens new avenues for chronotherapy targeting the CCL2-CCR2 axis in atherosclerosis treatment.

Mast cells (MCs), known for their roles in allergy and parasitic infections, are also pivotal in inflammation and fibrosis. CCL2 acts as an activator of MCs (85). Luo et al. (86) highlighted that early MC-fibroblast interaction and the stem cell factor/mast cell/CCL2/monocyte/macrophage axis are critical for initiating myocardial fibrosis. Additionally, hypoxia, which can be both a cause and a result of heart failure, induces CCL2 expression in both right ventricle (RV) and left ventricle (LV) of mice, potentially contributing to cardiac inflammation, fibrosis, and ventricular dysfunction (87).

## Pulmonary fibrotic diseases

Pulmonary inflammatory lesions tend to progress to fibrosis more consistently than in other organ systems, with CCL2 playing a significant role in this process. Pulmonary fibrosis is a multicellular phenomenon involving alveolar epithelial cells (AECs), recruited monocytes/macrophages, and fibroblasts. Several cell types, including AECs, produce CCL2, which promotes fibrosis via CCR2 activation (88). CCR2 signaling is therefore crucial for the development and progression of pulmonary fibrosis.

### Idiopathic pulmonary fibrosis

Idiopathic pulmonary fibrosis (IPF) is a progressive interstitial lung disease characterized by chronic inflammation, AEC damage, and excessive deposition of extracellular matrix proteins, with myofibroblasts acting as the main effector cells (89). In mouse models of bleomycin-induced pulmonary fibrosis, transcriptional analysis has shown a significant increase in CCL2 expression (90, 91). FoxF1, an endothelial transcription factor, has also been implicated in pulmonary fibrosis. *In vitro*, studies reveal that FoxF1-deficient endothelial cells enhance lung fibroblast proliferation, invasion, activation, and macrophage migration by secreting chemokines such as CCL2, collectively contributing to fibrosis (92). Moreover, IL-33 treatment increases CCL2 and CXCL2 production in *NFATc3^+/+^* macrophages, but not in *NFATc3^+/-^* mice, suggesting that NFATc3 regulates pulmonary fibrosis by regulating CCL2 and CXCL2 expression in macrophages (93).

Myofibroblasts play a central role in IPF pathogenesis. Single-cell RNA sequencing (scRNA-seq) data from IPF patients indicates that the CCL2/CCR2 axis is essential for M1 macrophage polarization (94). Pathogenesis varies among IPF subgroups, with ligand-receptor analysis suggesting a monocyte-macrophage chemotactic axis, potentially involving CCL2-CCR2, particularly in cilia-rich subgroups (95). Immunohistochemical analysis of human lung tissues has shown that activated IPF fibroblasts possess high contractile forces and produce abundant CCL2 (96), with the NF-κB signaling pathway contributing to CCL2 production and release in these fibroblasts (97). Another study on acute exacerbation of IPF found significantly higher CCL2 concentrations in bronchoalveolar lavage fluid compared to serum, indicating a localized inflammatory response (98).

### Radiation-induced pulmonary fibrosis

Radiation exposure to the lungs triggers a damage response, including the release of cytokines and chemotactic mediators, signaling the recruitment of immune cells for inflammation and wound healing. A recent study has reported significantly elevated CCL2 expression in macrophages of irradiated lungs, which potentially stimulates the fibrotic phenotype in lung fibroblasts (99). Following radiation, bone marrow-derived inflammatory monocytes migrate to the lungs, where CCR2^+^ monocyte-derived macrophages infiltrate and may play a critical role in the development of Radiation-induced pulmonary fibrosis (RIPF) (100).

### Other pulmonary fibrotic diseases

CCL2 also contributes to receptor-mediated pulmonary fibrosis progression. Adhesion G Protein-Coupled Receptor F5 (ADGRF5) is a key regulator of lung surfactant homeostasis in type II alveolar cells. Research has shown that ADGRF5 can regulate *CCL2* gene expression, maintaining its potential role in lung immune homeostasis. Disruption of ADGRF5 results in airway inflammation mediated by type 2 immune response and CCL2-induced inflammation (101). In a mouse model of pulmonary fibrosis, Leucine-Rich Repeat Kinase 2 (LRRK2) expression is significantly reduced in alveolar type II epithelial (ATII) cells, and LRRK2-deficient ATII cells exhibit an enhanced ability to recruit profibrotic macrophages via the CCL2/CCR2 axis. This recruitment triggers extensive macrophage-associated profibrotic responses and progressive pulmonary fibrosis (102).

Additionally, while IL-17 is known for its pro-inflammatory role in the intestine, it also contributes to parenchymal fibrosis in chronic pulmonary graft-versus-host disease (cpGVHD). Blocking IL-17A leads to reduced CCL2 expression in cpGVHD-related pulmonary fibrosis (103), though this effect is not observed in kidney diseases (104).

## Renal fibrotic diseases

During kidney injury, CCL2 recruits immune cells such as macrophages and T cells to the site of injury, activating them and triggering the release of additional inflammatory mediators. These mediators not only worsen kidney damage but also promote fibrosis. Proteomic analysis of an *in vitro* model of renal fibrosis identified CCL2 as a key factor contributing to collagen deposition in the kidney (105).

### Unilateral ureteral obstruction

Unilateral ureteral obstruction (UUO) can lead to renal interstitial fibrosis and is a potential precursor to chronic kidney disease (CKD), promoting extensive research into the underlying mechanisms. As mentioned earlier, activation of the Notch signaling pathway is known to upregulate CCL2 expression, contributing to fibrosis in organs like the liver (41). Recent animal studies have shown that Notch3 is newly expressed in damaged renal epithelium at early stages of CKD. Notably, systemic deficiency of Notch3 prevents leukocyte invasion and organ fibrosis, suggesting that targeting Notch3 may protect the epithelial epithelium and interrupt pro-inflammatory signaling, thereby alleviating kidney injury (106). Brandt et al. (107) utilized chimeric mice with Notch3 deficiency in hematopoietic cells and/or resident histiocytes to analyze renal fibrosis and inflammation following UUO. Their results indicated that a pro-inflammatory environment is characterized by upregulation of CCL2 and CCL5, which are regulated in a Notch3-dependent manner.

A recent study identified increased Twist1 expression in both the UUO mouse model and IgA nephropathy. Knockout of Twist1 in macrophages partially inhibited CCL2-mediated macrophage chemotaxis and suppressed M2 macrophage polarization, thereby mitigating fibrosis progression (108). Conversely, IL-15 has been shown to reduce CCL2 expression in the UUO mouse model, alleviating fibrosis and decreasing the likelihood of progression to CKD (109). Nicotinamide exhibits similar effects (110).

### Other renal fibrotic diseases

In CKD patients, angiopoietin-1 has been found to reduce the expression of chemokine CCL2 in fibrotic renal endothelial cells. In contrast, angiopoietin-2 induces CCL2 expression in endothelial cells, which promotes macrophage infiltration and increases apoptosis in fibrotic renal endothelial cells. This process negatively impacts the renal survival rate in CKD patients (111). Consistently, angiotensin-converting enzyme 2 (ACE2), which is inversely correlated with CCL2 expression, is strongly associated with CKD and is known to limit renal fibrosis (112). Another important factor is protease-activated receptor 2 (PAR2), which is involved in renal inflammation and fibrosis. Activation of PAR2 in cultured renal tubular epithelial cells triggers extracellular signal-regulated kinase signaling and CCL2 secretion, contributing to tubulointerstitial inflammation and fibrosis (113).

Abnormal immune system activation is also implicated in CKD development. Wilkening et al. (114) analyzed glomerular CCR2 expression in focal segmental glomerulosclerosis (FSGS) and demonstrated that macrophages expressing CCR2 contribute to kidney damage and fibrosis remodeling in conditions such as glomerulonephritis and diabetic nephropathy. In contrast, atypical chemokine receptor 2 (ACKR2), also known as D6, degrades CCL2, limiting the recruitment of immune cells and myofibroblasts to renal mesenchymal cells. This degradation inhibits renal inflammation and fibrosis remodeling in glomerulonephritis (115).

Kashyap et al. (116)reported that CCL2 deficiency protects against chronic kidney damage in a mouse model of renovascular hypertension caused by renal artery stenosis (RAS). The deficiency leads to reduced monocyte infiltration and lower expression of CCR2 and CD206, suggesting that CCL2 is a key mediator of kidney injury in renovascular hypertension.

## Bone marrow fibrotic diseases

Myelofibrosis (MF) is a prominent clinical feature observed in patients with myeloproliferative neoplasms (MPN). Prior research has established a correlation between the progression of MF and the aberrant expression of cytokines (117). Within the context of myeloproliferative disorders, CCL2 and its associated signaling pathways may play a critical role. CCL2 is thought to be involved in both the proliferation and differentiation of fibrosis-associated cells, thereby impacting the bone marrow microenvironment.

### MPN

A recent investigation revealed that among the MPN cohort, the proportion of CCR2^+^ cells is significantly associated with the severity of myelofibrosis in patients, and CCR2 expression on CD34^+^ cells correlates with higher-risk classifications in MF and the presence of circulating blast cells (118). Additionally, polymorphisms in the CCL2 gene have been shown to influence the bone marrow microenvironment in MPN, with homozygosity for the CCL2 rs1024611 SNP linked to diminished survival in individuals with primary myelofibrosis (119). The -2518 A/G SNP of CCL2 has emerged as a potential susceptibility marker for MPN and myelofibrosis (120).

### Systemic mastocytosis

As noted in the aforementioned myeloproliferative neoplasms, there is also a frequent elevation in the production of profibrotic and angiogenic cytokines in Systemic mastocytosis (SM), which contributes to alterations in the bone marrow microenvironment. A defining characteristic of SM is the abnormal accumulation of neoplastic MCs harboring the activating KIT mutation D816V within the bone marrow. Recent studies indicate that KIT D816V enhances CCL2 expression in abnormal MCs via the classical NF-κB signaling pathway. Furthermore, it has been demonstrated that CCL2 derived from MCs facilitates the migration of endothelial cells in SM, both *in vitro* and *in vivo*, thereby modifying the tumor microenvironment to favor fibrosis and angiogenesis. Serum CCL2 levels are markedly elevated in SM patients, with significantly higher concentrations observed in individuals with advanced SM compared to asymptomatic SM patients and those with cutaneous mastocytosis. Importantly, CCL2 levels exhibit only a moderate correlation with MCs infiltration in the bone marrow and instances of myelofibrosis (121).

### Acute myeloid leukemia

Literature indicates that the CCL2/CCR2 axis may be implicated in cell migration in acute AML. However, plasma CCL2 levels in Acute myeloid leukemia (AML) patients are reported to be lower than those in healthy donors (122). This finding contrasts with earlier observations made by Manzur et al. (123), highlighting the need for further investigation.

## Other fibrotic diseases

Fibrosis of the skin and internal organs is a hallmark of systemic sclerosis (SSc), with monocytes playing a crucial role in the skin’s inflammatory response (124). Studies have shown that CCL2 expression is significantly higher in SSc mouse models compared to normal mice (125), and CCL2 holds significant promise as a valuable biological marker for SSc (126). Additionally, knockout of STAT6 in SSc mouse models significantly reduces the expression of CCL2 and CD206, indicating that CCL2 is involved in the immune response that drives skin and organ fibrosis (127).

Experimental autoimmune orchitis (EAO) serves as an animal model for studying chronic testicular inflammation and fibrosis, replicating pathogenic changes similar to those seen in humans. During EAO, there is an increase in pro-inflammatory CCL2 expression, which coincides with leukocyte infiltration into the testicular parenchyma. Elevated levels of activin A are correlated with the severity of EAO, while high CCL2 levels mediate leukocyte transport and the recruitment of macrophages through its receptor, CCR2. These findings suggest that both CCR2 and activin A promote fibrosis in testicular inflammation by regulating macrophage function (128).

## CCL2/CCR2 axis as a diagnostic marker for fibrotic diseases

### Renal diseases

Urine is an easily obtainable sample that can reflect the severity of kidney disease, making it a focus for studying CCL2 levels as a diagnostic tool and for predicting prognosis. Urrego-Callejas et al. (129) included 120 systemic lupus erythematosus (SLE) patients and found that serum anti-C1Q antibody and urinary ceruloplasmin levels were associated with CCL2 activity in chronic injury. Similarly, in 125 patients with active lupus nephritis (LN), urinary CCL2 levels were positively correlated with interstitial inflammation, glomerulosclerosis, interstitial fibrosis, and tubular atrophy based on kidney biopsies (130). Standard LN therapies, like mycophenolate and rapamycin, improve glomerular sclerosis by downregulating CCL2 and reducing fibrosis-related proteins (131).

Studies have shown that excessive cisplatin administration induces renal interstitial fibrosis in C57BL/6 mice, and CCL2 serves as a marker of renal injury in this model (132). Additionally, high urinary CCL2 and low urinary epidermal growth factor (EGF) levels are associated with renal tubulointerstitial fibrosis (133), suggesting that urinary CCL2 could be a useful diagnostic marker for CKD patients.

Identifying early prognostic markers in high-risk renal transplant recipients can help optimize immunosuppressive therapy and improve outcomes. The ratio of CCL2 to creatinine (CCL2:Cr) has been shown to predict BK nephropathy; however, the diagnostic value of CCL2 for BKV infection in immunocompromised transplant recipients still needs further investigation (134).

### Cardiovascular disease

Macrophages drive vasculopathy changes in Takayasu arteritis (TA) through phenotypic transformation. Peripheral CCL2 levels fluctuate at different disease stages, suggesting CCL2 as a potential biomarker for disease activity (135). In human atria, markers of leukocyte infiltration and matrix degradation indicate severe inflammation—a key factor in atrial fibrillation—linked to CCL2 (136). In a study of 131 patients undergoing aortic valve replacement, CCL2 was identified as an inflammatory marker for aortic remodeling (137). In diabetic cardiomyopathy (DCM), both serum CCL2 and myocardial CCL2 mRNA levels are elevated (138).

### IBD

An interesting study of 33 children with IBD found that blood CCL2 levels were significantly higher in patients with CD compared to those with UC at all stages of the disease (139). This suggests that CCL2 could serve as a diagnostic biomarker for CD and help differentiate subtypes of pediatric IBD. However, it remains unclear if the same pattern is present in adult IBD patients. Given that both heart failure and IBD have immune-related pathogenesis, a study identified 34 genes associated with immune diseases, including CCL2, through Gene Ontology (GO) and Kyoto Encyclopedia of Genes and Genomes (KEGG) analysis (140).

### IPF

As an inflammation-related gene, *CCL2* may help predict the prognosis of IPF (141). While combined detection of CCL2, KL-6, and CXCL13 improves diagnostic accuracy for idiopathic interstitial pneumonia (IIP), it does not effectively differentiate among lung fibrosis diseases (142). Interestingly, serum CCL2 levels may help distinguish between IPF and fibrotic hypersensitivity pneumonitis (fHP), two conditions that are often difficult to differentiate (143).

## CCL2 as a therapeutic target for fibrotic diseases

Recent research has identified CCL2 as a key therapeutic target for treating fibrotic diseases across various organs. Its significance in fibrosis pathogenesis has led to the development of diverse treatment strategies, including traditional Chinese medicines (TCM), chemical agents, and application of nanotechnology. The broad potential of targeting CCL2 underscores the need for further research to fully leverage its role in creating effective antifibrotic therapies. CCR2 antagonists show significant potential for treating fibrotic diseases by specifically blocking CCR2 receptors. These antagonists have demonstrated positive effects in various conditions, including liver, lung, and renal fibrosis. Studies suggest that CCR2 antagonists reduce inflammatory cell infiltration, inhibit profibrotic gene expression, and slow fibrosis progression by modulating relevant signaling pathways.

### Liver diseases

In addition to the previously mentioned GILZ and SPHK1 that play a role in regulating CCL2 expression and thereby affecting liver fibrosis progression (54, 55), several pharmacological agents can also mitigate liver fibrosis by targeting the CCL2/CCR2 signaling pathway. Moreover, recent developments in nanotechnology have shown promise in the treatment of liver fibrosis through the modulation of CCL2 levels.

A recent investigation has introduced an innovative strategy involving the silencing of CCR2 through small interfering RNA (siCcr2) encapsulated within tetrahedron framework DNA nanostructure (tFNA) vehicle (tFNA-siCcr2). This method effectively overcomes the challenges associated with the *in vivo* efficacy of siCcr2 by facilitating targeted delivery to the liver, and resulting in improved therapeutic effects for liver fibrosis (144). Additionally, genetic engineering is being explored to create transgenic nano decoys that interfere with liver fibrosis. These decoys, engineered to overexpress CCR2 on their surface, can neutralize CCL2 levels. When loaded with curcumin and delivered to the liver, this combined therapy effectively reduces macrophage infiltration, offering promising therapeutic outcomes (145).

TCM formulations like Dahuang Zhizhu Pill, Tianhuang formula, and Fu-Gan-Wan have been shown to reduce the expression of CCL2 and its receptor CCR2 in the liver, particularly targeting CCR2^+^ macrophages (146–148). Similarly, Fuzheng Huayu (FZHY) modulates macrophage recruitment and polarization via CCL2 and CX3CL1, providing anti-inflammatory and antifibrotic effects. The Ganxianfang formula exhibits comparable benefits and may offer superior protective effects compared to FZHY (149). These findings suggest that TCM’s antifibrotic effects primarily result from inhibiting CCL2-induced macrophage recruitment to fibrotic sites, though the exact mechanisms—direct or indirect—remain unclear.Cenicriviroc (CVC) has gained attention for its therapeutic value in liver diseases. Krenkel et al. (150) conducted studies in NAFLD patients and C57BL/6 mice, demonstrating that CVC is effective and safe. CVC also enhances the therapeutic effect of fibroblast growth factor 21 on NASH, primarily by reducing liver fibrosis (151). In a mouse model of liver fibrosis induced by CCL4 and using Ccr2 knockout (*Ccr2^-/-^*) mice, Guo et al. (152) showed that CVC treatment was effective in both mice and human liver samples. Yu et al. (153) further demonstrated that CVC was effective in treating cholestatic liver injury in bile duct-ligated rats and Mdr2 (*Abcb4^-/-^*) mouse models. Additionally, Mbade et al. (154) found that CVC could prevent liver injury and steatosis in an alcoholic liver disease mouse model.

### Bile duct diseases

The CCL2/CCR2 axis’s chemotactic effect on monocytes and macrophages is a key driver of primary sclerosing cholangitis (PSC) progression. Studies have shown a positive correlation between serum CCL2 levels and fibrosis severity in primary biliary cholangitis (PBC) patients (155). In acute sclerosing cholangitis mouse models treated with the apoptosis-inhibiting BV6 inhibitor, both CVC treatment and CCR2 deletion were found to reduce macrophage accumulation, liver damage, and biliary fibrosis (156). Similarly, Reuveni et al. (157) observed improvements in primary biliary cholangitis models using *Cx3cr1^gfp/+^* and *Ccr2^-/-^Cx3cr1^gfp/+^* mice following intraperitoneal CVC injection.

### Intestinal diseases

In the treatment of IBD, agonists or inhibitors targeting specific factors or receptors have been developed. For example, SARI, commonly used as a colon cancer inhibitor, has been found to increase CCL2 production when deficient, whereas knocking out CCR2 can block this effect, thereby reducing colitis symptoms (158). Dimethyl itaconate decreases CCL2 production in epithelial cells, reduces macrophage recruitment to the tumor microenvironment, alleviates the hyperinflammatory state of UC, and lowers the risk of colitis-associated cancer (159).

Several other drugs directly target the CCL2/CCR2 axis. Luteolin and homoharringtonine both exhibit strong anti-inflammatory effects by inhibiting NF-κB signaling and reducing CCL2 production in colonic tissues (160, 161). Nobiletin reduces CCL2 expression and collagen deposition in colitis-induced mice (162), while berberine and geniposide show similar effects in chronic colitis (163, 164).

### Pulmonary diseases

Excessive inflammation in silicosis is triggered by silica exposure. Research has shown that the Caspase-1 inhibitor VX-765 reduces the infiltration of inflammatory M1 alveolar macrophages and decreases CCL2 expression, thereby mitigating the inflammatory response (165). TAS-115, a novel polytyrosine kinase inhibitor, has been found to inhibit the phosphorylation of the macrophage colony-stimulating factor receptor c-FMS in mouse bone marrow-derived macrophages, both *in vivo* and *in vitro*. This inhibition reduces CCL2 production, highlighting CCL2 as a key molecule in pulmonary fibrosis development (166).

### Pancreatic diseases

Oxidative stress is a major pathway in pancreatitis pathogenesis. Hydroxytyrosol has anti-inflammatory and antioxidant effects, reducing CCL2 release in pancreatic and colon tissues (167). Additionally, the classic TCM formulation known as Dahuang Chaihu Decoction has been shown to lower the levels of CCL2 in the pancreas, which in turn reduces macrophage infiltration and fibrosis associated with CP (168).

### Renal diseases

In a glomerulosclerosis model, the use of a CCR2 antagonist (CCR2A) can inhibit renal macrophage accumulation and enhance the effects of conventional treatments like angiotensin-converting enzyme inhibitors (ACEi) (169). CKD often leads to complications such as salt-sensitive (SS) hypertension. Daily intraperitoneal administration of a CCR2 antagonist (RS 102895) during active disease stages has been shown to reduce renal leukocyte infiltration, kidney injury, and hypertension incidence (170). Additionally, bladder outlet obstruction (BOO) causes bladder remodeling, affecting its structure and function. Wang et al. (171) demonstrated that treatment with a CCR2 antagonist (RS504393) may offer a therapeutic strategy for managing bladder remodeling in such conditions.

## Clinical applications of CCL2/CCR2 antagonists

**Figure 3:**
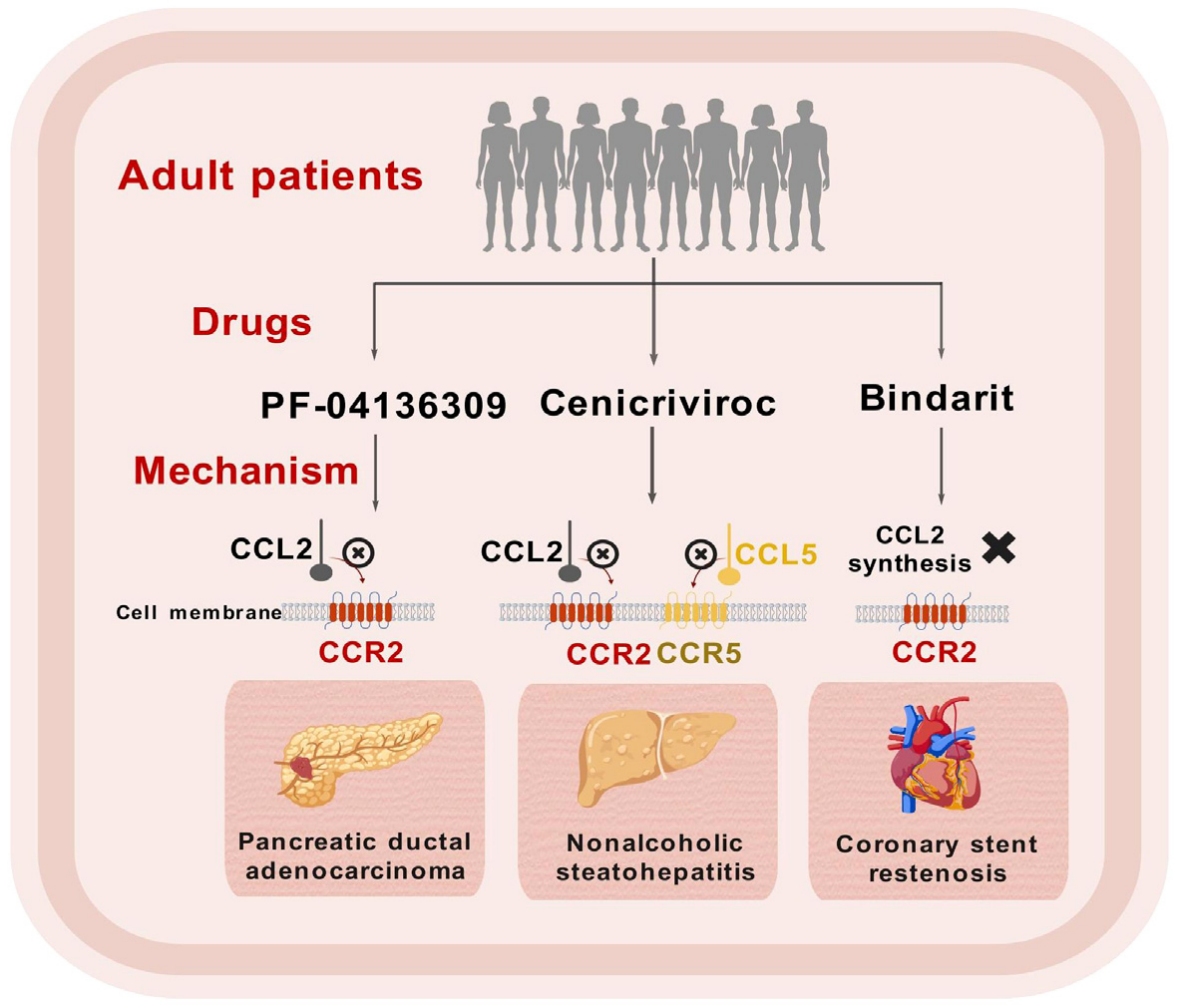
Schematic representation of clinical trials for CCL2/CCR2 inhibitors. This figure was created with biogdp.com.

In recent years, a considerable number of clinical studies focusing on adult patients have been conducted to explore the effects of CCL2/CCR2 antagonists (**Figure 3**). Currently, significant advancements have been made in clinical research concerning the treatment of NASH-related liver fibrosis using CVC, with clinical trials advancing to Phase III. It is widely acknowledged that the medication shows good tolerability, with a safety profile similar to that of a placebo (172). Initial results from ongoing trials indicate that CVC may offer additional benefits for patients suffering from advanced fibrosis (173, 174). However, the combination of CVC with Tropifexor did not yield improved therapeutic efficacy (175). Although the Phase III clinical trial demonstrated good therapeutic efficacy, it still lacks histological data from clinical studies to confirm its efficacy in the treatment of liver fibrosis (176).

Primary sclerosing cholangitis (PSC) is a chronic cholestatic condition that can result in liver fibrosis. A multicenter clinical trial conducted with adult patients diagnosed with PSC revealed that after 24 weeks of treatment with CVC, there was a moderate decrease in the surrogate marker of serum alkaline phosphatase (ALP), and the therapy was well tolerated by the participants (177).

In addition to fibrotic diseases, there has been notable advancement in clinical trials involving CCL2/CCR2 antagonists for other conditions, including pancreatic ductal adenocarcinoma (PDAC) (178, 179) and coronary stent restenosis (180). Future exploration of larger clinical studies in these domains is warranted (**Table 1**).

**Table 1:** Clinical trials of CCR2/CCL2 antagonists in treating inflammatory and fibrotic diseases.

Drug(s)	Mechanism	Disease	Research Stage	Efficacy and safety	Trial identification	Authors (year) [reference]
CVC; Tropifexor (TXR)	TXR, a potent nonbile acid FXR agonist	NASH	IIb	Combination therapy safety similar to monotherapy; no significant improvement in efficacy.	NCT03517540	Anstee et al. (2023) (175)
CVC	CCR2/5 dual antagonist	NASH	II	While overall safety remains satisfactory, the patient’s condition has not improved significantly.	NCT03059446	.Francque et al. (2024) (172)
			IIb	Safe and improves liver fibrosis in some patients.	NCT02217475	Friedman et al. (2018) (173)
			III	Good efficacy and tolerability; significant effect on advanced fibrosis.	NCT02217475	Ratziu et al. (2020) (174)
			III	Safe, but did not meet histological efficacy criteria.	NCT03028740	Anstee et al. (2024) (176)
		PSC	II	Demonstrated good efficacy and safety.	NCT02653625	Eksteen et al. (2021) (177)
PF-04136309	PF-04136309(CCR2 antagonist) in combination with FOLFIRINOX	PDAC	Ib	Good tolerance, most patients experienced remission.	NCT01413022	Nywening et al. (2016) (178)
PF-04136309	PF-04136309 in combination with nab-paclitaxel/gemcitabine	PDAC	Ib	High incidence of lung toxicity; combination therapy showed no better efficacy than monotherapy.	NCT02732938	Noel et al. (2020) (179)
Bindarit	Selective inhibitor of CCL2	Coronary stent restenosis	II	Showed therapeutic effect with good tolerability; did not reach a primary endpoint.	NCT01269242	Colombo et al. (2016) (180)

CVC, Cenicriviroc; NASH, Nonalcoholic steatohepatitis; PSC, Primary sclerosing cholangitis; PDAC, Pancreatic ductal adenocarcinoma.

## Conclusions and future perspectives

**Figure 4:**
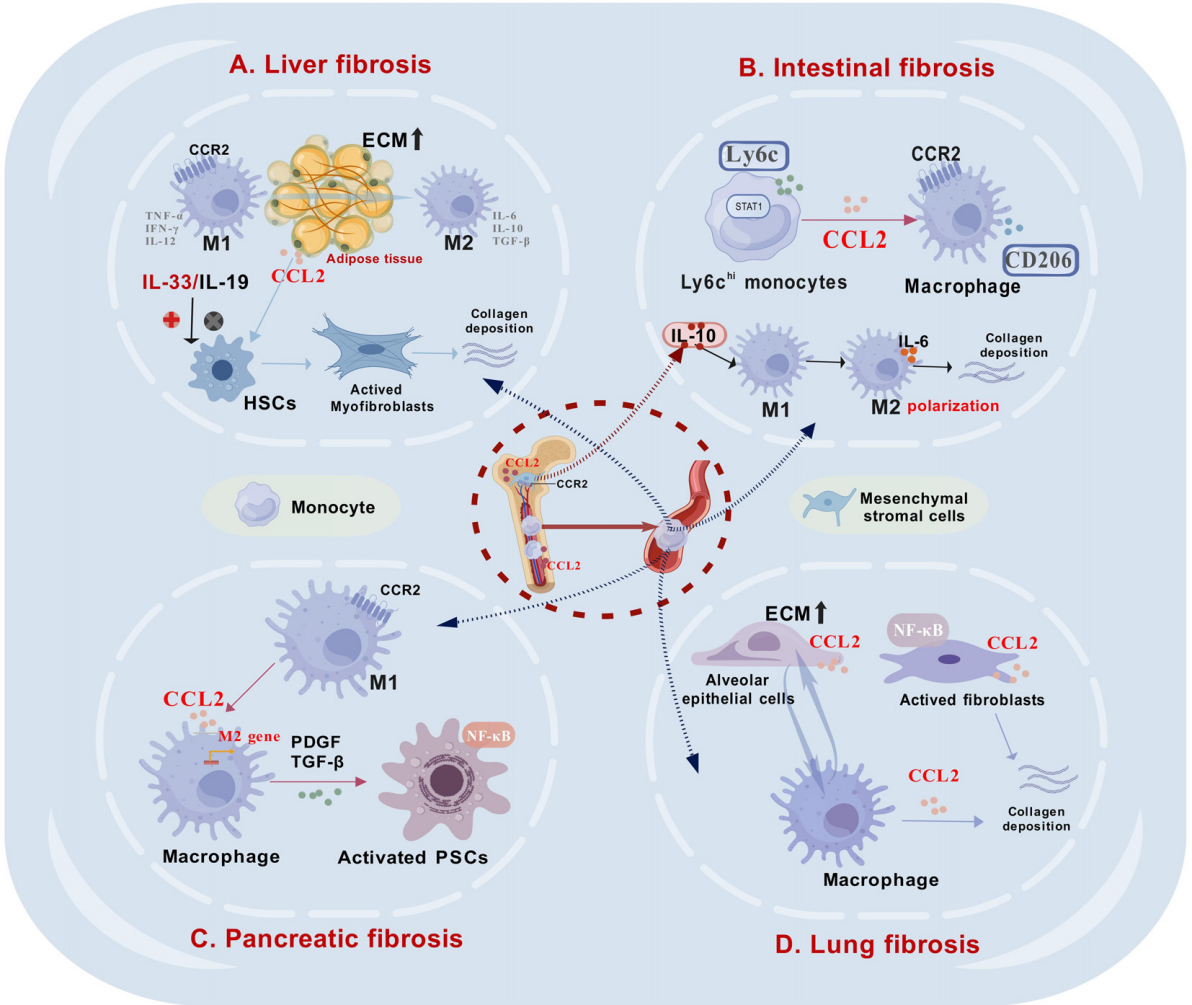
The role of CCL2 in mediating intercellular interactions within fibrotic tissues in various organs. **(A)**. In liver fibrosis, CCL2 plays a critical role by recruiting macrophages to the fibrotic regions and facilitating their M2 polarization, which subsequently activates hepatic stellate cells (HSCs) and promotes collagen deposition. **(B)**. In intestinal fibrosis, Ly6C^hi^ monocytes are predominant, and an increase in their proportion significantly enhances the expression of CCL2. Bone marrow-derived mesenchymal stromal cells (BM-MSCs) can release IL-10, which facilitates the M2 polarization of intestinal macrophages, subsequently contributing to the development of fibrosis. **(C)**. In pancreatic fibrosis, M2 macrophages secrete pro-fibrotic factors that activate pancreatic stellate cells (PSCs), a process similar to that in liver fibrosis. **(D)**. In lung fibrosis, alveolar epithelial cells (AECs) exhibit increased CCL2 production, facilitating macrophage recruitment to fibrotic regions. Additionally, activated fibroblasts release significant amounts of CCL2 via NF-κB signaling, contributing to collagen accumulation. This figure was created with biogdp.com.

Recent research underscores the crucial role of the CCL2/CCR2 axis in the development and progression of inflammatory and fibrotic diseases across various organs (**Table 2**). While the mechanisms by which CCL2 mediates cell interactions differ slightly between organs (**Figure 4**), its primary role in most fibrotic conditions is to recruit macrophages to sites of tissue damage. An exception is viral hepatitis, where CCL2 expression is downregulated in liver cells, contributing to an immunosuppressive environment that allows viral persistence, a key early step in the disease’s pathogenesis.

**Table 2:** The role of CCL2/CCR2 axis in organ fibrosis.

Disease	Main role of CCL2/CCR2 axis in fibrosis	Reference
Hepatic fibrosis	The CCL2/CCR2 axis not only attracts chemotactic macrophages to the liver but also promotes M1 polarization of macrophages, activating hepatic stellate cells (HSCs) via inflammatory factors like IL-1β and TNF-α and signaling pathways such as NF-κB.	(39, 41, 43)
Intestinal fibrosis	In intestinal tissues, the CCL2/CCR2 axis attracts chemotactic monocytes/macrophages to fibrotic regions, induces M2 polarization, and participates in inflammation-mediated fibrosis through factors like IL-6 and IL-17.	(57, 59, 60, 62, 65, 67, 69)
Cardiovascular fibrosis	The CCL2/CCR2 axis recruit leukocytes to inflammatory areas and promotes monocyte differentiation into macrophages, where M1-type macrophages aid in myocardial healing.	(77, 78)
Pulmonary fibrosis	In pulmonary fibrosis, alveolar epithelial cells and macrophages are primary sources of CCL2. They release IL-4 and IL-13 upon inflammatory stimulation, which activates myofibroblasts and promotes fibrosis progression.	(94–97)
Renal fibrosis	In kidney injury, various cells such as renal tubular epithelial cells, macrophages, and fibroblasts release CCL2. Binding to CCR2 recruits chemotactic monocytes to the damaged site, releasing TGF-β and IL-6.	(106–108)

In pancreatic fibrosis, the role of the CCL2/CCR2 axis is less understood. It remains unclear whether CCL2 is the primary chemokine driving macrophage infiltration in the pancreas or which genes trigger its activation during fibrosis. Recent evidence suggests that SPHK1 is critical in promoting fibrosis in CP models (181). Given SPHK1’s regulatory effect on CCL2 in liver fibrosis, it is worth investigating if a similar mechanism occurs in SPHK1-induced pancreatic fibrosis.

Another area needing exploration is the role of CCL2 in pain associated with fibrotic diseases. Emerging studies have linked CCL2 to pancreatic tumor growth, neural invasion, and pancreatic cancer-related pain (182, 183). Since pain is a common, debilitating symptom in many inflammatory and fibrotic conditions, such as CP (184), understanding how CCL2 contributes to neural invasion and pain mechanisms could reveal new research and treatment avenues.

The therapeutic potential of targeting the CCL2/CCR2 axis is promising. Various strategies, including natural compounds, chemical agents, and traditional Chinese medicine, have been explored to modulate this pathway in treating fibrotic diseases, highlighting its clinical translation potential. Beyond inflammatory and fibrotic conditions, targeting CCL2 could also enhance cancer treatment, particularly immunotherapy in cancers such as esophageal squamous cell carcinoma (185, 186) and breast cancer (187). Circulating CCL2 levels may also serve as biomarkers for predicting the onset, progression, and prognosis of various cancers, including liver and prostate cancers. Further research is needed to determine if CCL2 has similar predictive value in other cancers and its potential to improve chemotherapy outcomes.

Clinical trials of CCL2/CCR2 antagonists are ongoing for various diseases. In conclusion, recent studies investigating the role of CCR2 antagonists in treating fibrotic diseases have shown promising safety profiles, with the majority of participants reporting significant symptom relief. However, there is an urgent necessity for more sophisticated assessment methodologies to rigorously evaluate the effectiveness of CCL2/CCR2 inhibitors in various fibrotic conditions. Notwithstanding these challenges, CCL2 is anticipated to undergo further evaluation as a viable therapeutic target for a broader spectrum of organ-related diseases, potentially providing patients with innovative and effective treatment alternatives.
